# Development of Lectin Modified Fluorescent Magnetic Particles for Highly Sensitive Detection of Glycoconjugates

**DOI:** 10.3390/s21165512

**Published:** 2021-08-17

**Authors:** Yoshio Suzuki

**Affiliations:** Health and Medical Research Institute, National Institute of Advanced Industrial Science and Technology (AIST), 1-1-1 Higashi, Tsukuba, Ibaraki 305-8566, Japan; suzuki-yoshio@aist.go.jp; Tel.: +81-29-861-6122

**Keywords:** fluorescence, lectin, magnetic particles, optical sensors

## Abstract

I conducted this study to develop an improved method for glycome detection using fluorescent magnetic beads, whose surfaces were modified using lectins, for the highly sensitive detection of saccharides or glycoproteins via fluorescence quenching using a novel fluorescence emitter and quencher pair. The emitter (Cy3 fluorophore) was incorporated into magnetic beads, and a fluorescence quencher (cyanopyranyl group) was bound to glycomes via covalent bonding. The fluorescence intensities of fluorescent magnetic beads containing lectins decreased specifically in the presence of glycomes, which was a result of fluorescence quenching from Cy3 to cyanopyranyl groups due to the formation of a stable complex between lectins and glycome. Fluorescence intensities were plotted as a function of glycoprotein concentration, and good linear relationships were observed. This method enabled the fluorescent reading-out of a series of lectin-glycome interactions on the basis of recognition selectivity and affinity of immobilized lectins without tedious washing processes. Moreover, a simple profiling process was performed using this assay for diverse glycoconjugates, which not only included simple saccharides but also glycoproteins and glycome in cell lysates. These results clearly indicate that the combination of magnetic beads with the novel emitter-quencher pair enabled the highly sensitive detection of lectin-glycome interactions.

## 1. Introduction

The glycome, which is the repertoire of glycans, such as oligosaccharides, glycolipids, and glycoproteins, produced by individual biological systems such as cells, tissues, and organisms, has been widely studied to modulate numerous physiological and pathological states [1,2]. Glycosylation is an important posttranslational modification of proteins that profoundly affects various chemical and biological processes such as apoptosis, angiogenesis, and microbial recognition [3,4,5]. Glycosylation on cell surfaces is essential for biological processes, which include growth and adhesion of living cells, immune responses, and the maintenance of diverse living systems. Several studies have indicated that disorders in biological processes contribute to several diseases such as diabetes and cancers [6,7,8,9,10,11]. Moreover, cancer-related structural modifications of glycans, such as sialylation, fucosylation, and polylactosamine elongation, are caused due to cellular perniciousness transformation [12,13,14,15]. Presently, mining of the glycome for cancer-associated biomarkers represents a new paradigm for cancer diagnosis and prognosis, and the detection of glycoconjugates as biomarkers of biological events is not only important for the advancement of basic scientific research but also for diagnostic applications. Additionally, model systems such as self-assembled monolayers and gold glyconanoparticles have been widely used to study carbohydrate-protein binding events [16,17,18]. However, the detection limits of previous assays are poor, and more sensitive analytical systems are needed for the detection of carbohydrate-protein interactions due to the inherent diversity, complexity, and heterogeneity of glycan structures that make glycome analysis particularly challenging. 

Recently, optical biosensors based on nanomaterials have been designed and synthesized for the analysis of various biological substances based on fluorescence spectroscopic methods [19,20,21,22]. To improve the performance of sensors, fluorescent materials that exhibit a spectral response upon binding to chemical and biological substances need to be developed. This concept generally produces a high-throughput, highly sensitive, and selective analysis of target molecules.

I previously developed fluorescent sensors for the detection of various saccharides based on lectin-saccharide interactions using fluorescent magnetic beads [23]. Lectins are proteins, and bind to specific glycome. Magnetic beads are widely used for the biological study from the view point of quick and easy separation to produce low background, high purity and high sensitivity results [24,25]. To take advantage of the merits of lectins and magnetic beads, fluorescent analysis of the glycomes was performed with the use of a FRET-based sensing system. However, the photostability of the sensing system is poor due to the poor lifespan of dansyl moiety immobilized on magnetic beads, and the fluorescence intensities are dependent on pH, which may lead to incorrect results and does not allow the detection of physiological levels of target glycome in long-term measurements.

**Figure 1:**
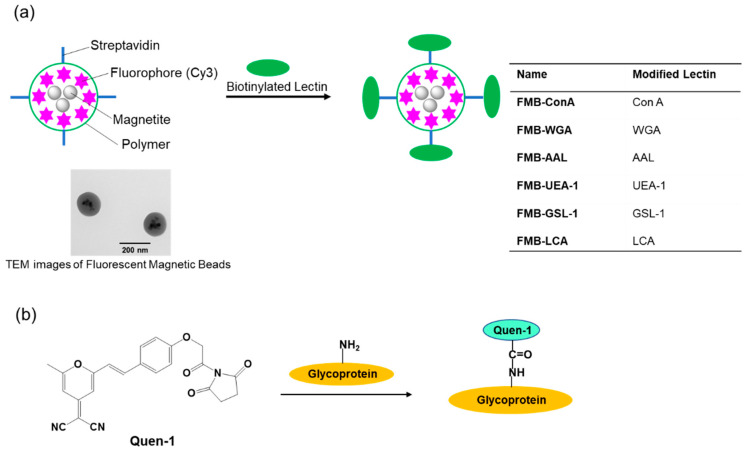
Schematic representation of the immobilization of lectins onto the surface of fluorescent magnetic beads (designated as FMB-Lectin; Lectin: Con A, WGA, AAL, UEA-1, GSL-1, or LCA) (**a**) and the labeling reaction between fluorescent quenchers and glycoproteins (**b**).

Therefore, I conducted this study to develop an improved method for glycome detection that utilizes the properties of nanometer-sized fluorescent magnetic beads, whose surfaces were modified using lectins via avidin-biotin interactions (FMB-Lectin), as shown in Figure 1a. Cy3 fluorophore was used as the fluorescent emitter, and cyanopyranyl group was used as the fluorescent quencher in the sensing system. Fluorophores (Cy3) were incorporated into magnetic beads, which were surrounded by poly-glycidyl methacrylate and prevented the influence of solution pH on the fluorescence intensity of FMB-Lectin. In contrast, the cyanopyranyl group, which is a fluorescent quencher of Cy3, was introduced into the glycome via covalent bonding under mild and rapid reaction conditions (Quen-Glycan). The lectin-glycoconjugate interactions between molecules in a homogenous solution system were investigated based on the fluorescence quenching efficiency of Cy3 in FMB-Lectin, and the cyanopyranyl group of glycome as used for glycome detection based on the decreased fluorescence-changing activity of the two compounds. I investigated the specificity, sensitivity, stability, reusability, and glycome detection capability of this improved glycome assay in human cell lysates. The experimental results clearly indicated that this assay may be widely applicable for the detection of lectin-glycome interactions, and it may enable long-term longitudinal measurements of the glycome at physiological levels. This supports the administration of precise doses of therapeutic glycome for several important human diseases as well as the accurate monitoring of glycome levels.

## 2. Materials and Methods

### 2.1. General Information

All chemicals used were of analytical grade, and they were all purchased from Tokyo Chemical Industry (Tokyo, Japan), Wako Pure Chemical Industries, Ltd. (Osaka, Japan), or Sigma-Aldrich. Fluorescent magnetic beads modified with streptavidin were purchased from Tamagawa Seiki Co., Ltd. (Nagano, Japan). Absorption spectra were recorded at 25 °C using a UV/visible spectrophotometer (V-670, JASCO Corporation, Hachioji, Japan), and fluorescence spectra were recorded at 25 °C using a JASCO FP-6500 fluorophotometer(JASCO Corporation, Hachioji, Japan).

### 2.2. Modification of Glycomes Using Fluorescence Quenchers 

(E)-2-(2-(4-(2-(2,5-dioxopyrrolidin-1-yl)-2-oxoethoxy)styryl)-6-methyl-4H-pyran-4-ylidene)malononitrile (Quen-1) was labeled to glycoproteins as follows: The activated-esterized compound Quen-1 was dissolved in 50 μL dimethylsulfoxide and the mixture was added to 500 μL of PBS buffer (pH 7.4) in the presence of 1.0 mg glycoprotein. The reaction mixture was stirred at 4 °C for 12 h, and it was purified via gel permeation chromatography (eluent: PBS buffer). Based on the UV-VIS spectra, the first fraction, which was yellow in color, was identified as a glycoprotein modified using Quen-1. 

### 2.3. Labeling of Lectins onto Fluorescent Magnetic Beads 

The optimal experimental conditions for the immobilization of biotinylated lectins onto fluorescent streptavidin magnetic beads were as follows: 1 mg of fluorescent streptavidin magnetic beads (50 µL of 20 mg/mL beads) were added to each of the microtubes, and subsequently, PBS buffer solution was added. After spinning down, the beads were magnetically separated, and PBS buffer solution was then added. After the addition of biotinylated lectin (100 μL) at a concentration of 0.5 mg/mL in PBS, the reaction mixture was stored at 4 °C for 1 h. After spinning down, the reacted magnetic beads were magnetically separated, and they were then washed with HEPES buffer solution (pH 7.9) twice. After addition of preservative buffer, the fluorescent magnetic beads modified using lectins were stored at 4 °C.

### 2.4. Reaction of Fluorescent Magnetic Beads Labeled Using Lectin with Glycoproteins

Fluorescent magnetic beads labeled with lectin were added to HEPES buffer solution (pH 7.0) at a final concentration of 1.0 μg/100 μL. After the addition of 50 μL of glycoprotein to 50 μL of fluorescent magnetic beads, the reaction mixture was incubated. After spin down, magnetic separation, and brief washing, the fluorescence spectra of reacted fluorescent magnetic beads were recorded to analyze the amount of lectin-glycoprotein complexes.

### 2.5. Release of Glycoproteins from Fluorescent Magnetic Beads by the Addition of Carbohydrate Inhibitors

Carbohydrate inhibitors were added to the solution of fluorescent magnetic beads labeled using lectin and glycoprotein in PBS buffer solution (pH 7.0), and the changes in fluorescence intensities were monitored over time. The carbohydrate inhibitors used were as follows: α-lactose (0.1 M), melibiose (0.1 M), d-mannose (0.1 M), N-acetyl-d-glucosamine (0.05 M), and α-L-fucose (0.05 M).

### 2.6. Modification of Glycomes in Cell Lysates Using Fluorescence Quenchers 

The activated-esterized compound Quen-1 was dissolved in 50 μL dimethylsulfoxide and the mixture was added to 50 μL of cell lysates. The reaction mixture was stirred at 4 °C for 12 h, and was used for the measurement using lectin-modified fluorescent magnetic beads without further purification. 

## 3. Results and Discussion

**Figure 2:**
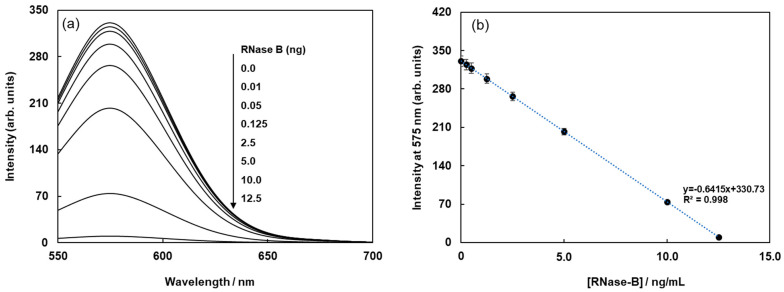
Fluorescence spectra of FMB-Con A before and after the addition of RNase-B modified using Quen-I (**a**), and plots of the fluorescence intensities of FMB-Con A at 575 nm against the concentrations of RNase-B modified using Quen-I (**b**). [FMB-Con A] = 10 μg/mL; [RNase-B] = 0~12.5 ng/mL; Solvent, PBS buffer solution, pH 7.0; Excitation wavelength, 550 nm.

The biotinylated lectin concanavalin A (Con-A) was immobilized on the surface of streptavidin-based magnetic beads containing a fluorophore (Cy3) via avidin-biotin interactions, as shown in Figure 1a. Glycoproteins were labeled with (E)-2-(2-(4-(2-(2,5-dioxopyrrolidin-1-yl)-2-oxoethoxy)styryl)-6-methyl-4H-pyran-4-ylidene)malononitrile(Quen-1) through amino groups in glycoproteins and activated ester groups in Quen-1, as shown in Figure 1b, and they were then reacted with lectin-modified fluorescent magnetic beads. As a typical example, ribonuclease B (RNase B), which has a rich mannoside branch, was used as the glycoprotein, and it was labeled using Quen-1. Fluorescent magnetic beads were modified using Con A (FMB-Con A), and they were used to detect RNase B. Figure 2a shows the fluorescence spectral changes in FMB-ConA before and after the addition of various concentrations of RNase B. Fluorescence intensities decreased with an increase in the amount of RNase B due to the high affinity of Con A for the mannoside branch of RNase B, which was because the Cy3 fluorophores in magnetic beads were in close proximity to the cyanopyranyl group in RNase B due to the high affinity of Con A to mannoside branch in RNase B. The cyanopyranyl group in RNase B was the fluorescence quencher against Cy3 fluorophore in magnetic beads due to spectral overlaps of the emission spectrum in Cy3 and absorption spectrum in cyanopyranyl moiety, which caused the fluorescence quenching of FMB-ConA using RNase B modified by Quen-1. 

The fluorescence intensities of FMB-ConA at 560 nm were plotted as a function of RNase B concentration, and the results are shown in Figure 2b. The plot revealed a good linear relationship between fluorescence intensity and RNase B concentration. The limit of detection for RNase B was 0.25 ng/mL, and the signal-to-noise ratio was 3.0. 

**Figure 3:**
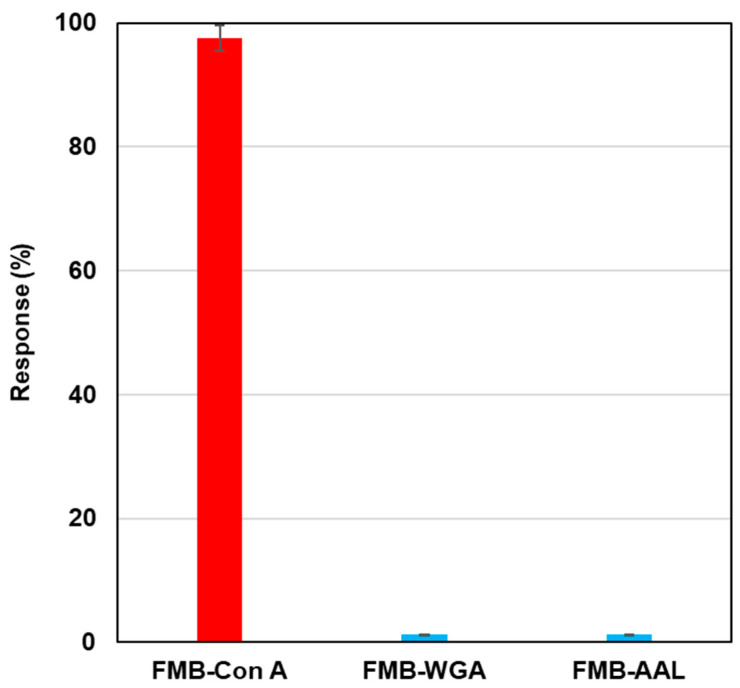
Fluorescence responses of FMB-Con A, FMB-WGA, and FMB-AAL after the addition of RNase-B modified using quenchers. [FMB-Con A] = [FMB-WGA] = [FMB-AAL] = 10 μg/mL; [RNase-B] = 12.5 ng/mL; Solvent; HEPES buffer solution, pH 7.0; Excitation wavelength; 550 nm; Monitored wavelength; 575 nm; Response (%) = (I_0_ − I)/I_0_ × 100 (I_0_: fluorescence intensities of blank, I: fluorescence intensities after the addition of saccharides).

To investigate the selectivity of lectin-labeled fluorescent magnetic beads against various glycoproteins, biotinylated lectins, such as Con A, wheat germ agglutinin (WGA), and aleuria aurantia lectin (AAL), were modified on the surface of streptavidin-based fluorescent magnetic beads (FMB-WGA and FMB-AAL), and fluorescence intensities were monitored after the addition of RNase B modified by Quen-1. Figure 3 shows the results of selectivity tests. FMB-Con A showed a high affinity towards α-Man, and its fluorescence response was highest, and FMB-WGA and FMB-AAL showed no responses towards RNase B, because WGA has a high affinity to the GlcNAc family and AAL has a high affinity for fucose. These lectins have no affinities toward mannoside branches. Based on these results, the fluorescence responses of fluorescent magnetic beads indicated the high affinities between lectins and glycoproteins, and the selectivity of lectin-glycome interactions could be monitored with the sensing system.

**Figure 4:**
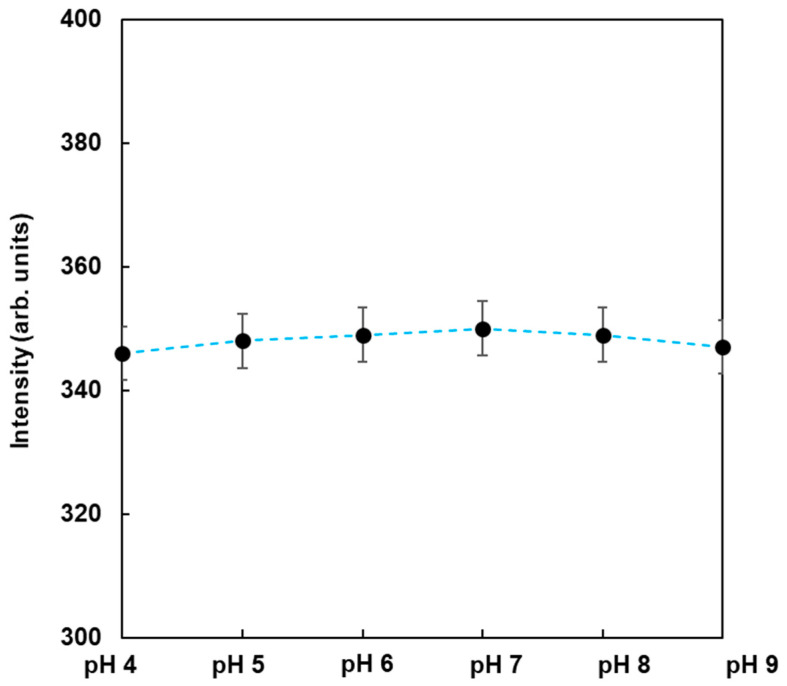
Fluorescence intensities of FMB-Con A in the buffer at different pH values. [FMB-Con A] = 10 μg/mL; Excitation wavelength, 550 nm.

To observe the dependence of pH on the fluorescence from FMB-ConA, the fluorescence intensities of FMB-ConA were monitored at different pH values ranging from 4 to 9. The data are shown in Figure 4. As a result, the fluorescence intensities were nearly unchanged when the pH was in the range of 4 to 9, whereas the fluorescent magnetic beads in the author’s previous study were affected due to pH [23]. This was due to the fact that the fluorophores in this study were encapsulated in magnetic beads and surrounded by poly GMA, which contributed to pH independence. On the other hand, fluorophores in a previous study were immobilized on the surface of magnetic beads, and their fluorescence intensities were affected due to pH. As a result, FMB-ConA satisfied the experimental conditions for the in vitro analysis of lectin-glycoprotein interactions, and this method can be applied to investigate real samples in the field.

**Figure 5:**
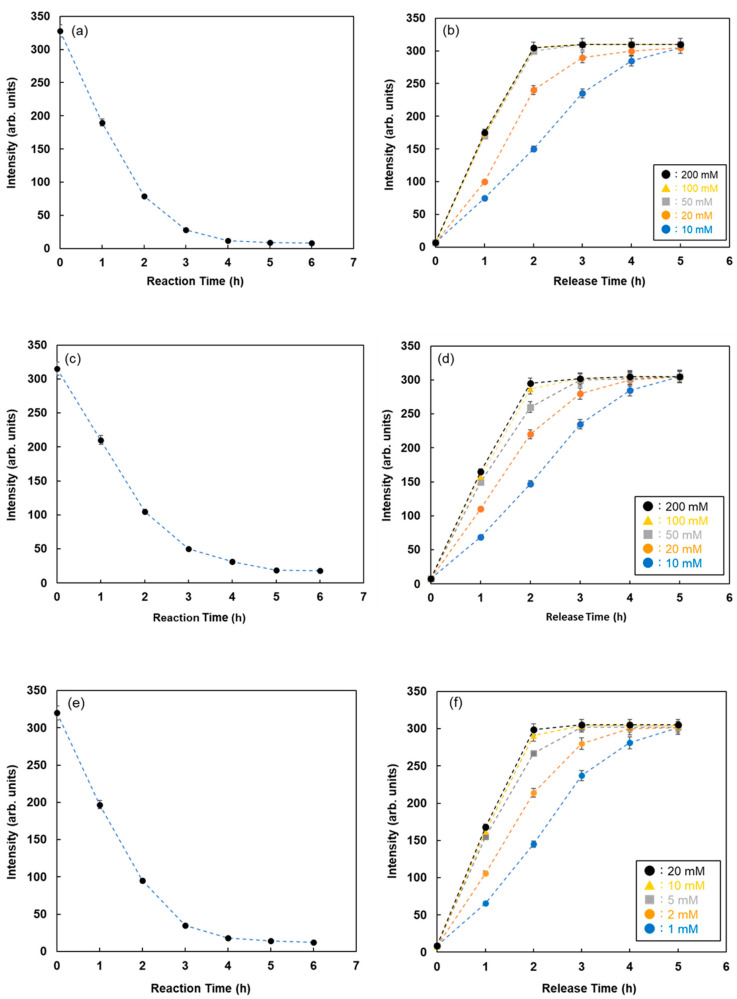
Fluorescence intensities of FMB-Con A with time after the addition of RNase-B modified using Quen-I (**a**) and after the addition of various concentrations of maltose as the inhibitor sugar (**b**). Fluorescence intensities of FMB-WGA with time after the addition of ovalbumin modified using Quen-I (**c**) and after the addition of various concentrations of N-acetyl-D-glucosamine as the inhibitor sugar (**d**). Fluorescence intensities of FMB-UEA-1 with time after the addition of mucin modified using Quen-I (**e**) and after the addition of various concentrations of α-L-Fucose as the inhibitor sugar (**f**). [FMB-Con A] = [FMB-WGA] = [FMB-UEA-1] =10 μg/mL; [RNase-B] = [Ovalbumin] = [Mucin] =10.0 ng/mL; Solvent, PBS buffer solution, pH 7.0; Fluorescence measured at 575 nm; Excitation wavelength, 550 nm.

The reaction time between FMB-Con A and RNase B attached to Quen-1 was investigated by monitoring the fluorescence intensities of FMB-Con A, and the results are shown in Figure 5a. The fluorescence intensities of FMB-Con A decreased with time after the addition of RNase B, and the decrease in fluorescence intensity stopped after 6 h. It is known that the reaction of lectins with glycoproteins takes approximately 6 h or overnight, which is consistent with the above results.

Inhibitor sugars, such as D-glucose and α-L-fucose, were used for the recovery of glycoproteins adsorbed on the lectin column. To take advantage of this phenomenon, the glycoproteins adsorbed on fluorescent magnetic beads were recovered, and the elution process was monitored by assessing the changes in fluorescence intensities with time after the addition of inhibitor sugars. Figure 5b shows the changes in fluorescence intensity with time for RNase B adsorbed on FMB-Con A after the addition of various concentrations of maltose. The fluorescence intensities increased with time due to the release of RNase B from the surface of FMB-Con A, and the release rate increased as a function of maltose concentration. As a result, this method is available for visualizing the lectin-glycoprotein interactions and releasing glycoproteins from lectins on the surface of fluorescent magnetic beads, which makes it easier to detect the reaction conditions for lectin-glycoprotein interactions.

To investigate the lectin-glycoprotein interactions and release of glycoproteins from fluorescent magnetic beads by the addition of inhibitor sugars using this method, WGA and Ulex europaeus agglutinin I (UEA-1) were immobilized on the surface of fluorescent magnetic beads (FMB-WGA and FMB-UEA-1, respectively). The interactions between FMB-WGA and Ovalbumin and FMB-UEA-1 and Mucin, and the effect of the addition of inhibitor sugars, such as N-acetyl-D-glucosamine for FMB-WGA and α-L-fucose for FMB-UEA-1, were monitored by observing the changes in fluorescence intensities over time. The results are shown in Figure 5c–f. The emission intensities after the addition of glycoproteins decreased with time due to the interactions between lectins on the surface of magnetic beads and glycoproteins. Moreover, the addition of inhibitor sugars caused the recovery of fluorescence emissions due to the release of glycoproteins from lectins on the surface of fluorescent magnetic beads. As a result, the detection and reaction of various glycoproteins with fluorescent magnetic beads containing lectins were successfully achieved using fluorescence spectrometry. The combination of energy donor and acceptor molecules and lectins played an important role in variations in fluorescence intensity and the highly sensitive detection of glycoproteins.

**Figure 6:**
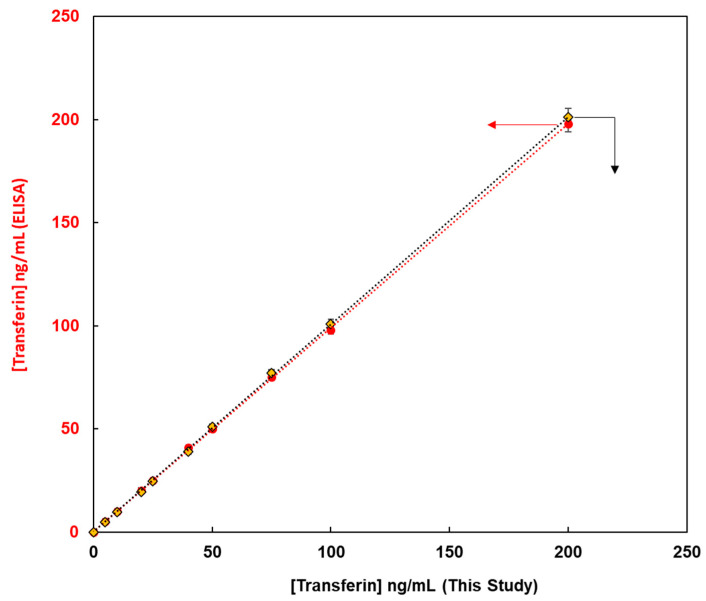
The relationship between transferin concentrations obtained using this method on X-axis and those obtained using ELISA on Y-axis.

This method, constructed from the magnetic bead-based assay for the detection of glycoproteins, was compared with enzyme-linked immunosorbent assay (ELISA) methods from the viewpoint of quantitative analysis of glycoproteins. Fluorescent magnetic beads were modified using lens culinaris agglutinin (LCA) lectin via avidin-biotin bonding for the detection of human transferrin (FMB-LCA), whereas commercially available ELISA kits were used for the detection of human transferrin. Using these analytical methods, the unknown concentrations of human transferrin samples were detected, and the observed concentrations from calibration graphs were compared. Figure 6 depicts the transferrin concentrations using this paper’s method on the X-axis and those via ELISA on the Y-axis. Thus, a good linear relationship was observed, and glycoproteins were successfully evaluated. Moreover, the limit of detection for this paper’s method was 0.1 ng/mL, whereas that for ELISA was 4.6 ng/mL. Glycoprotein analysis in this study proved more sensitive than the conventional ELISA method, and it is a qualitative, quantitative analytical method that can be widely used as a high-throughput method for the measurement of glycoprotein levels in human biofluids.

**Figure 7:**
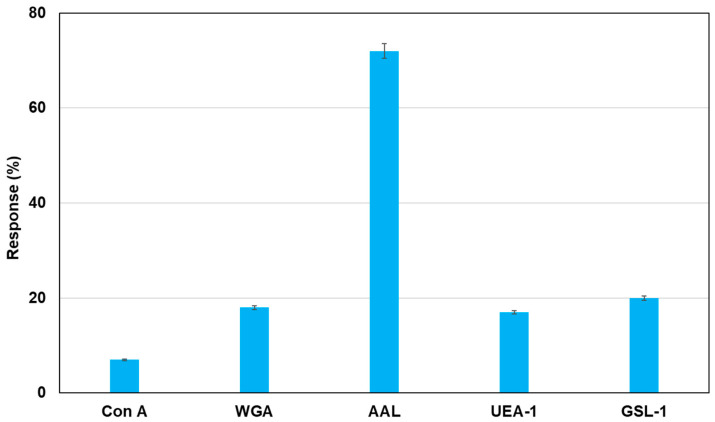
Detection of glycome patterns in cell lysates from MCF-7 using fluorescent magnetic beads. The bar graph of fluorescent response following the addition of mammalian cell lines (MCF-7: 10,000 cells/µL). [FMB-Con A] = [FMB-WGA] = [FMB-AAL] = [FMB-UEA-1] = [FMB-GSL-1] = 10 μg/mL; Fluorescence measured at 575 nm; Excitation wavelength, 550 nm.

**Figure 8:**
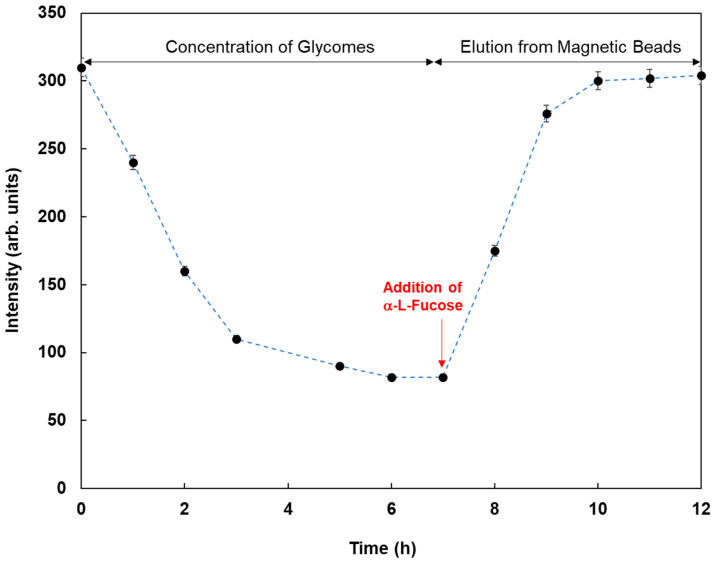
Fluorescence intensities of FMB-AAL with time by the reaction with cell lysates from MCF-7 before and after the addition of α-L-Fucose as the inhibitor sugar. [FMB-Con A] = 10 μg/mL; [α-L-Fucose] = 20.0 mM; Fluorescence measured at 575 nm; Excitation wavelength, 550 nm.

As an application of this method using real samples, cell lysates originating from MCF-7 were profiled. Fluorescent magnetic beads were modified using Con A, WGA, AAL, UEA-1, and GSL-1, and they were reacted with cell lysates from MCF-7, which were monitored using fluorescence spectrometry. Figure 7 shows the fluorescence responses by the reaction of various fluorescent magnetic beads with cell lysates from MCF-7. FMB-AAL showed a strong response, which was consistent with the presence of a higher number of fucosylated O-linked sugar chains in MCF-7 cells than in other cells [26]. Moreover, the reactions between FMB-AAL, which indicated the strongest responses, and cell lysates from MCF-7 were monitored before and after addition of α-L-fucose as inhibitor sugar, and the results are shown in Figure 8. The emission intensities after the addition of cell lysates decreased with time due to the interactions between lectins on the surface of magnetic beads and glycomes in cell lysates. The addition of inhibitor sugars caused the recovery of fluorescence emissions due to the release of glycomes from lectins on the surface of fluorescent magnetic beads. As a result, this method could successfully profile cell lines based on the characteristic difference in the sugar components, and information on the amount of glycome in human cells was obtained using this method. 

## 4. Conclusions

In conclusion, fluorescent magnetic beads containing lectins were synthesized, and lectin-saccharide and lectin-glycoprotein interactions were detected with the use of “on-off”-based sensing systems. Moreover, the sensing system could fluorescently respond to the types and concentrations of sugars or glycoproteins using a high-throughput and convenient sensing of various saccharide derivatives. The present lectin beads are very promising for many objectives, particularly for the throughput analysis of all glycoconjugates in cell lines and organisms. Future studies will focus on investigating possible indicators for the detection of glycome and other biological substances in combination with various analytical methods.

## Data Availability

Data presented in this study are available on request due from the corresponding author. The data are not publicly available due to privacy.
